# Simultaneous supplementation of encapsulated amino acids and capped dietary starch levels improved growth performance of broiler chickens fed by low-protein diet

**DOI:** 10.1016/j.psj.2026.107217

**Published:** 2026-06-02

**Authors:** Ramin Shahabi, Hassan Darmani Kuhi, Mohammad Roostaie Ali-Mehr, Houshang Lotfollahian

**Affiliations:** a Department of Animal. Science, Faculty of Agricultural Sciences, University of Guilan, Rasht, Iran; b Agricultural Research, Education, and Extension Organization (AREEO), Animal Science Research Institute, Karaj, Iran

**Keywords:** Broiler chicken, Encapsulated amino acids, Low protein diet, starch

## Abstract

This experiment was designed to investigate the effect of encapsulated amino acids supplementation and capped dietary starch levels on the growth performance of broiler chicks fed low-protein diets. Dietary crude protein (CP) was gradually reduced from 200 g/kg (CP20, a high-protein baseline diet) to 180 g/kg (CP18) and 160 g/kg (CP16). To ensure that essential amino acid requirements were met despite these reductions, encapsulated amino acids were supplemented to the diets. A 2 × 2 factorial design was implemented, involving two starch levels (capped and uncapped) and two glycine levels (with and without encapsulated glycine supplementation). Each dietary treatment was replicated 6 times with 11 male Ross 308 broiler chicks in each replicate, totaling 396 birds fed from 15 to 36 days of age. All dietary treatments were isoenergetic (3000 kcal AMEn/kg) with 10.9 g/kg standardized ileal digestible (SID) Lysine. The CP18 diet had no significant effect on weight gain (WG) and feed intake (FI), but feed conversion ratio (FCR) significantly increased by 3.24% compared to the CP20 diet (P < 0.0001). The CP16 diet USL+Gly significantly reduced WG by 10.19% and increased FCR by 7.87% (P < 0.0001). Capped dietary starch levels in the low protein (LP) diets significantly increased WG compared to USL treatments, with no significant difference from the CP20 treatment. There was no significant difference between the FCR of capped starch level (CSL) diets and the CP18 diet. Relative abdominal fat weight increased by 4% CP reduction (P = 0.0338), but was not affected by dietary starch levels. Dietary AMEn significantly increased with increasing dietary starch level (P < 0.0001). It was concluded that supplementing LP diets with encapsulated amino acids did not mitigate the reduction in growth performance in LP diets. To achieve similar WG as observed in the HP diet, it is essential to cap dietary starch levels while incorporating slow-release amino acid supplements into the LP diet.

## Introduction

Global chicken meat production is projected to double by 2050 to meet the rapidly increasing demand for animal protein (Selle et al., 2022), which places substantial pressure on the poultry industry to implement sustainable production systems that ensure feed ingredient supply, reduce production costs, mitigate environmental pollution, and maintain bird health and welfare. Protein constitutes the most expensive component of poultry feed, with soybean meal serving as the dominant source globally. However, the rising global price of soybean meal, exacerbated by high import costs and customs fees, poses a significant economic burden on importing countries. Furthermore, the escalating demand for this ingredient drives deforestation and places severe environmental pressure on primary producer nations (Selle et al., 2022). Therefore, these challenges pose a multi-dimensional framework for managing and decreasing soybean meal consumption in poultry production. Precision poultry nutrition plays a pivotal role in this transition by improving nutrient utilization efficiency while reducing nitrogen losses (Selle et al., 2022, 2023).

One of the most promising nutritional strategies is the replacement of crude protein (CP)-based feeding systems with amino acid–balanced low protein (LP) diets (Moss et al., 2021; Wu et al., 2021; Wu and Li, 2022). Low-protein diets reduce dependence on soybean meal in broiler chickens. By implementing these diets, producers can also decrease nitrogen (N) excretion, leading to improved environmental sustainability (Hilliar et al., 2019; Cappelaere et al., 2021; Alfonso-Avila et al., 2022; Liang et al., 2023). However, dietary CP reduction beyond 2–3% has consistently been reported to have adverse effects on weight gain (WG), feed conversion ratio (FCR), and body fat deposition (Fancher and Jensen, 1989; Aletor et al., 2000; Namroud et al., 2008; Darsi et al., 2012; Belloir et al., 2017; van Harn et al., 2019; Chrystal et al., 2020a, Chrystal et al., 2020b; Hilliar et al., 2020; Greenhalgh et al., 2022; Sadeghi et al., 2024). Low-protein diets are characterized by increased inclusion of free amino acids (approximately five times), naturally elevated dietary starch-to-protein ratios, and reduced concentrations of nonessential amino acids, particularly glycine (Selle et al., 2022).

Studies have reported that dietary free amino acids (AAs) are absorbed approximately four times faster than protein-bound AAs, which disrupts the synchronism of AA absorption and post-absorptive AA balance (Yen et al., 2004; Liu et al., 2013; Morales et al., 2019; Zamani et al., 2021). This asynchrony leads to increased oxidative deamination, higher blood ammonia concentration, and ammonia toxicity, which are key factors that negatively affect WG in low-protein (LP) diets (Schreurs et al., 1997; Nolles et al., 2009; Selle et al., 2015, 2022, 2023). Recent research suggests that using slow-release forms of AAs offers a promising approach to improving LP diet efficiency by enhancing postprandial AA bioequivalency. The use of encapsulated AAs in a solid fat matrix in pig, laying hen, and broiler chicken nutrition has demonstrated several benefits: slow AA release in the gut without reducing bioavailability, improved post-absorptive AA balance, increased AA utilization efficiency without compromising production performance, and a delayed and synchronized AA release compared to protein-bound AAs in the diet (Piva et al., 1997; Prandini et al., 2013; Sun et al., 2020a, Sun et al., 2020b; Erwan et al., 2020; Liu et al., 2024).

To mitigate the compromised growth performance observed in broilers fed LP diets, it is crucial to consider three interrelated factors during feed formulation. First, there is reduced bioequivalency between free amino acids and protein-bound amino acids, which can hinder their effectiveness. Second, there is impaired metabolic synchrony between glucose and amino acid supply at the sites of protein synthesis, which is essential for optimal growth. Finally, insufficient availability of glycine limits the capacity for uric acid synthesis and ammonia detoxification. Collectively, these issues lead to reduced amino acid utilization efficiency, ultimately compromising the growth performance of broilers on LP diets.

In LP diet studies, the primary objective is typically to achieve growth performance equivalent to that of a conventional high-protein diet. The standard high-protein diet is routinely supplemented with uncoated amino acids, representing the industry norm. In the current study, encapsulated amino acids were used to supplement the gradually CP reduced diets in order to compare the effects of the experimental treatments on alleviating the performance reduction caused by CP reduction with the standard high-protein diet. Moreover, capped and uncapped dietary starch level with and without encapsulated glycine supplement in 4% CP reduced diets were experimented to evaluate effect of dietary starch and glycine levels on growth performance of the broiler chickens.

## Materials and methods

All procedures were approved by the Research Integrity and Ethics Administration of the University of Guilan (IR.GUILAN.REC.1403.186).

### Free amino acids and encapsulation

This study employed three types of uncoated amino acids (DL-methionine, L-lysine-HCL, and L-threonine) for supplementing the standard high-protein diet, alongside nine kinds of encapsulated amino acids (Encapsulated DL-methionine, L-lysine-HCL, L-tryptophan, L-threonine, L-valine, L-isoleucine, L-arginine, L-histidine, and L-glycine) to address amino acid deficiencies in the low-protein diets, aligning with the experimental design and nutritional requirements of broiler chickens (Table 2). Feed-grade encapsulated DL-methionine, L-lysine-HCL, and L-tryptophan were procured from Golbar Navid Bahar Co. (Tehran, Iran), while other feed-grade amino acids, specifically L-threonine, L-valine, L-isoleucine, L-arginine, and L-histidine, were sourced from CJ BIO (Seoul, Korea), and L-glycine was obtained from Shanghai Chemex Group Ltd. (Shanghai, China). Amino acids were encapsulated using palm fat according to Sahraei-Belverdy et al. (2019), achieving coating values of 50-58% palm fat matrix, consistent with similar studies in poultry nutrition experiments (Sun et al., 2020a, Sun et al., 2020b; Liu et al., 2024). The final prepared encapsulated amino acids had the following specific purities: L-lysine 45%, DL-methionine 55%, L-threonine 49.25%, L-valine 48.25%, L-isoleucine 45%, L-arginine 45.25%, L-tryptophan 45%, L-histidine 50%, and L-glycine 49.75%.

### Experimental design and bird

A total of 450 one-day-old male Ross 308 broiler chicks were obtained from a commercial hatchery. Birds were fed a common maize–soybean meal starter diet (220 g/kg CP, 2900 kcal/kg AMEn) from day 0 to day 15 post-hatch. On day 15, 396 birds with uniform body weight were selected and randomly allocated to experimental treatments. Initial body weight was not significantly different between all treatments. The 6 dietary treatment was offered to 6 replicate pens with 11 birds per pen from day 15 to day 36 post-hatch. Six experimental corn–soybean meal–based diets were formulated to be isoenergetic (3000 kcal/kg AMEn) and iso-digestible-lysine (10.9 g/kg SID Lys), while differing in CP concentration, dietary starch level, and glycine level.

A standard high-protein diet containing 20% crude protein (CP20) was supplemented with uncoated amino acids including L-lysine, DL-methionine, and L-threonine. To assess the impact of a slow-release (encapsulated) form of amino acids in LP diets, encapsulated amino acids—L-lysine, DL-methionine, L-threonine, L-valine, L-isoleucine, L-arginine, L-tryptophan, L-histidine, and L-glycine—were used to meet the amino acid requirements of birds fed LP diets. Dietary CP was gradually reduced from 20% (CP20) to 18% (CP18) and then to 16%, with the latter treatment including an uncapped starch level and glycine supplementation (USL+Gly). To assess the effect of glycine levels in the LP diets, a CP16 diet with an uncapped starch level but without encapsulated glycine (USL-Gly) was included. In LP diets, starch levels naturally increase. To evaluate the impact of capping dietary starch in these diets, starch was capped in two treatments to match the starch level of the standard CP20 diet. One capped-starch treatment included encapsulated glycine (CSL+Gly), while the other did not (CSL-Gly). The main effects of starch level and glycine level, along with their interaction, were evaluated using a 2 × 2 factorial design based on data from the USL+Gly, USL-Gly, CSL+Gly, and CSL-Gly treatments in the low-protein (CP16) diets. The dietary treatments were as follows:

1. CP20: Standard control diet containing 200 g/kg CP supplemented with uncoated AAs
2. CP18: Low-protein diet containing 180 g/kg CP, supplemented with encapsulated AAs
3. CP16 USL+Gly: 160 g/kg CP, supplemented with encapsulated AAs, uncapped starch level (USL), with glycine supplementation
4. CP16 USL–Gly: 160 g/kg CP, uncapped starch level, supplemented with encapsulated AAs, without glycine supplementation
5. CP16 CSL+Gly: 160 g/kg CP, capped starch level, supplemented with encapsulated AAs, with glycine supplementation
6. CP16 CSL–Gly: 160 g/kg CP, capped starch level (CSL), supplemented with encapsulated AAs, without glycine supplementation

Uncapped starch level in diets reflects naturally elevated starch concentrations associated with CP reduction, whereas CSL in diets was formulated to achieve starch concentrations comparable to the CP20 control diet. In non-glycine-supplemented diets, the SID Gly+Ser to Lys ratio was reduced below recommended levels (Rostagno et al., 2024) to evaluate glycine limitation under LP conditions (Table 1).

**Table 1:** Dietary treatments characteristics (corn–soybean meal-based diets with 3000 kcal/kg AMEn and 10.90 g/kg digestible lysine).

		Estimated dietary concentrations		Analyzed dietary concentrations		
Dietary treatment^1^		Crude protein (g/kg)	Starch (g/kg)	Starch (g/kg)	Crude protein (g/kg)	Starch/Protein ratio
CP20		200	352	326.4	204.0	1.58
CP18		180	396	355.8	182.5	1.95
CP16	USL+Gly	160	438	419.8	156.1	2.69
	USL-Gly	160	435	412.7	160.1	2.58
CP16	CSL+Gly	160	352	313.1	164.6	1.90
	CSL-Gly	160	352	320.9	155.4	2.07

1 CP20: Standard control diet containing 200 g/kg CP; CP18: Low-protein diet containing 180 g/kg CP; CP16 USL+Gly: 160 g/kg CP, uncapped starch level, with glycine supplementation; CP16 USL–Gly: 160 g/kg CP, uncapped starch level, without glycine supplementation; CP16 CSL+Gly: 160 g/kg CP, capped starch level, with glycine supplementation; CP16 CSL–Gly: 160 g/kg CP, capped starch level, without glycine supplementation; USL: uncapped starch level; CSL: capped starch

### Bird management

Birds were housed in floor pens (1.6 m × 0.8 m × 1 m), equipped with 3 nipple drinkers and a tube feeder. Birds had ad libitum access to feed and water. Lighting was provided at 23L:1D from days 0–3 and 16L:8D thereafter. Ambient temperature was maintained at 32°C during the first week, gradually reduced to 22 ± 1°C by week 3, and maintained thereafter.

### Diet formulation and preparation

Diets were formulated based on corn–soybean meal to supply 10.9 g/kg SID Lys and 3000 kcal/kg AMEn in all dietary treatments (Table 2, Table 3). Essential amino acids were formulated relative to lysine according to the ideal amino acid ratios proposed by Wu (2014). The Gly+Ser to Lys ratio was 140 as suggested by Rostagno et al. (2024) for the 17-35 days of rearing period. Dietary electrolyte balance was maintained at 240 mEq/kg across all treatments. All LP diets (CP18 and all CP16 treatments) were supplemented with encapsulated amino acids, whereas the CP20 diet was supplemented with equivalent quantities of uncoated crystalline amino acids. In CSL diets, sand used as an inert filler to limit dietary corn and consequently starch level (Greenhalgh et. al, 2020). To avoid thermal damage to the fat matrix of encapsulated amino acids, all experimental diets were prepared and offered in mash form. Acid-insoluble ash (AIA; Celite; 15 g/kg) was included in all diets as an indigestible marker to assess starch and protein apparent digestibility in distal jejunum and distal ileum. All diets contained equivalent amounts of palm fat to standardize the energy contribution from the encapsulation matrix.

**Table 2:** Ingredients of the experimental diets^1^.

Ingredients (g/kg)	CP20	CP18	CP16 USL+Gly	CP16 USL-Gly	CP16 CSL+Gly	CP16 CSL-Gly
Sand	-	-	13.83	6.99	75.5	69.87
AIA	15	15	15	15	15	15
Corn	537	611	682	683	553	552
Soy Meal	357	288	191	206	217	232
Soy Oil	24.6	11.1	2.2	-	45.4	43.7
Palm fat	24.5	15.538	-	5.995	1.378	6.272
MCP	14.08	14.7	15.65	15.46	15.8	15.65
Calcium Carbonate	14.29	14.45	14.8	14.8	14.6	14.6
Vit-Min Supplement^2^	3.5	3.5	3.5	3.5	3.5	3.5
Choline Chloride	0.9	0.9	0.9	0.9	0.9	0.9
NaCl	2.6	1.6	1	1	1	1
NaHCO3	2.18	3.64	4.53	4.50	4.60	4.57
K2CO3	-	1.65	5.02	4.34	4.54	3.87
L Lys (%45)	0.83 (79%)	4.968	10.11	9.255	9.276	8.420
DL Met (%55)	2.54 (99%)	6.124	7.855	7.51	8.076	7.745
L Thr (%49.25)	0.73 (99%)	3.26	5.98	5.5	5.82	5.350
L Val (%48.25)	-	2.12	5.545	4.94	5.4	4.800
L Ile (%45)	-	1.23	4.9	4.26	4.53	3.900
L Arg (%49.25)	-	0.97	6.72	5.75	5.95	5.759
L Trp (%45)	-	-	1.07	0.89	0.9	0.724
L His (%50)	-	-	0.5	0.16	0.46	0.120
L Gly (%49.75)	-	-	7.64	-	7.12	-

1 CP20: Standard control diet containing 200 g/kg CP; CP18: Low-protein diet containing 180 g/kg CP; CP16 USL+Gly: 160 g/kg CP, uncapped starch level, with glycine supplementation; CP16 USL–Gly: 160 g/kg CP, uncapped starch level, without glycine supplementation; CP16 CSL+Gly: 160 g/kg CP, capped starch level, with glycine supplementation; CP16 CSL–Gly: 160 g/kg CP, capped starch level, without glycine supplementation; USL: uncapped starch level; CSL: capped starch

2 Provided the following (per kg of diet): vitamin A (trans-retinyl acetate), 10,000 IU; vitamin D_3_ (chole-calciferol), 2,000 IU; vitamin E (all-rac-tocopherol acetate), 20 IU; vitamin K (bisulfate menadione complex), 3 mg; riboflavin, 5 mg; pantothenic acid (d-calcium pantothenate), 10 mg; nicotinic acid, 30 mg; pyridoxine (pyridoxine HCl), 3 mg; thiamin (thiamin mononitrat e), 1 mg; vitamin B12 (cyanocobalamin), 12 μg; d-biotin, 0.15 mg; choline (choline chloride), 300 mg; folic acid, 0.5 mg; Se (Na_2_SeO_3_), 0.1 mg; I (KI), 2.0 mg; Cu (CuSO_4_5H_2_O), 10 mg; Fe (FeSO_4_7H_2_O), 30 mg; Mn (MnSO_4_· H2O), 100 mg; Zn (ZnO), 100 mg; and ethoxyquin, 110 mg.

**Table 3:** Nutrient specifications of the experimental diets^1^.

Analyse	CP20	CP18	CP16 USL+Gly	CP16 USL-Gly	CP16 CSL+Gly	CP16 CSL-Gly
AMEn	3000	3000	3000	3000	3000	3000
Crude protein	200	180	160	160	160	160
Starch	352	396	438	435	352	352
Starch/Protein ratio	1.76	2.20	2.74	2.72	2.20	2.20
Starch/SID Lys	32	36	40	40	32	32
Crude fat	74	62	52	52	93	91
Crude fiber	33	32	28	29	27	28
Ca	8.8	8.8	8.8	8.8	8.8	8.8
Ava. P	4.4	4.4	4.4	4.4	4.4	4.4
Na	1.8	1.8	1.8	1.8	1.8	1.8
K	8.7	8.2	8.7	8.2	8.2	8.2
Cl	2.3	2	2.3	1.9	2	2
DEB	240	240	240	240	240	240
SID^2^ amino acids						
Lys	10.9	10.9	10.9	10.9	10.9	10.9
Met	5.4	5.65	6.07	5.99	6.12	6.05
M+C	8.18	8.18	8.18	8.18	8.18	8.18
Thr	7.63	7.63	7.63	7.63	7.63	7.63
Trp	2.2	1.86	1.85	1.85	1.85	1.85
Arg	13.24	11.77	11.77	11.77	11.77	11.77
Val	8.79	8.72	8.72	8.72	8.72	8.72
Ile	8.09	7.55	7.52	7.52	7.52	7.52
His	5.12	4.5	3.82	3.82	3.82	3.82
Gly+Ser	17.40	15.28	15.26	12.36	15.26	12.52

1 CP20: Standard control diet containing 200 g/kg CP; CP18: Low-protein diet containing 180 g/kg CP; CP16 USL+Gly: 160 g/kg CP, uncapped starch level, with glycine supplementation; CP16 USL–Gly: 160 g/kg CP, uncapped starch level, without glycine supplementation; CP16 CSL+Gly: 160 g/kg CP, capped starch level, with glycine supplementation; CP16 CSL–Gly: 160 g/kg CP, capped starch level, without glycine supplementation; USL: uncapped starch level; CSL: capped starch

2 Standardized ileal digestible.

### Growth performance

Body weight (BW) and feed intake (FI) were recorded on days 15 and 36 on a pen basis, and WG, FI and FCR as calculated for the experimental period.

### Energy and nitrogen utilization

Excreta were collected from each pen on days 33 and 34 by spread a plastic sheet on top of the litter, oven-dried at 80°C for 24 h, and ground. Gross energy of diets and excreta was determined using an adiabatic bomb calorimeter (PAAR 1261, PAAR Instrument Company, Illinois, USA). Nitrogen content in diets and excreta was quantified using a nitrogen analyzer (FOSS Kjeltec™ 2300, FOSS Analytical AB, Höganäs, Sweden). Nitrogen retention values were derived using the following equation (Scott & Hall, 1998):

Apparent nitrogen retention = 1 – [(Marker diet / Marker excreta) × (N excreta / N diet)] × 100

Nutritional parameters assessed included apparent metabolizable energy (AME), metabolizable-to-gross energy ratios (ME:GE), nitrogen retention, and nitrogen-corrected AME (AMEn). The ME:GE ratios were calculated as AME divided by the gross energy of the corresponding diets. The AME values were calculated on a DM basis from the following equation (Stefanello et al., 2016):

AMEdiet(kcal/kgDM)=GEdiet(kcal/kgDM)−[GEexcreta(kcal/kgDM)×(Markerdiet/Markerexcreta)]

AMEndiet(kcal/kgDM)=AMEdiet−(NR×K)

K=8.73kcal/gNretained

NR(%)=Ndiet−[(Markerdiet/Markerexcreta)×Nexcreta]

N-corrected AME (AMEn kcal/kg DM basis) values were calculated by correcting N retention to zero using the factor of 8.73 kcal/g N retained in the body (Hill, 1958).

### Blood biochemistry

On day 35, brachial vein blood samples (2.5 mL) were collected from two randomly selected birds from each pen using heparinized vacutainers during active feeding. Samples were centrifuged at 3,000 × g for 10 minutes (4°C), pooled by pen, and stored at -80°C until analysis. Blood parameters, including triglycerides (TG), low-density lipoprotein (LDL), high-density lipoprotein cholesterol (HDL-C), total protein (TP), uric acid (UA), urea, aspartate aminotransferase (AST), and alanine aminotransferase (ALT), were assessed by colorimetric method (Mindray BS-200, Mindray, Guangdong, China).

### Carcass traits and digestive dynamics

At day 36, two birds with closest body weight to the average of pen were selected and euthanized. Weights of breast, thigh, liver, gizzard plus proventriculus, pancreas, and abdominal fat were recorded. Following the opening of the abdominal cavity and isolation of the small intestine, the digestive contents from the distal half of the jejunum and ileum were gently and completely expelled to prepare for subsequent sample collection. The jejunum was precisely defined as the segment extending from the end of the duodenal loop to Meckel’s diverticulum, while the ileum was delineated as the region from Meckel’s diverticulum to the ileocecal junction. Digesta samples were then carefully collected from the area situated posterior to the midpoint of each respective segment. Subsequently, samples obtained from the distal jejunum (DJ), alongside those from the distal ileum (DI), were separately pooled within each experimental pen. Digesta was mixed, freeze dried, and ground to determine the apparent digestibility coefficients of starch and protein. Starch content was determined in diet and digesta samples using the spectrophotometer (UV-M51, BEL Photonics, São Paulo, Brazil) method described by Clegg (1956). The apparent digestibility coefficients for starch and crude protein (N × 6.25) were calculated using the following standard equation:

$$Digestibility\;coefficient=\left({\left(Nutrient/AIA\right)}_{diet}-{\left(Nutrient/AIA\right)}_{digesta}\right)/{\left(Nutrient/AIA\right)}_{diet}$$

Daily nutrient disappearance rates (g/bird/day) for starch and protein (N) were calculated using the equation:

Disappearancerate(g/bird/day)=feedintake(g/day)×dietarynutrient(g/kg)×digestibilitycoefficient

### Statistical analysis

Data were analyzed using the GLM procedure of SAS software (SAS Institute, version 9.1, 2002, Cary, NC, USA) according to a completely randomized design (CRD) for data recorded from treatments CP20, CP18, CP16 (CP16 USL+gly), CP16 USL-gly, CP16 CSL+gly and CP16 CSL-gly. To evaluate the main effects of starch level, glycine levels, and their interaction, data recorded from CP16 USL+gly, CP16 USL-gly, CP16 CSL+gly and CP16 CSL-gly were analyzed in a 2 × 2 factorial arrangement by GLM procedure. Means were compared by Tukey test, with differences considered significant at P< 0.05 and tending to significance at P< 0.1.

## Results

### Growth performance

Growth performance results presented in Table 4. Broiler WG was significantly influenced by CP reduction (P < 0.0001). Crude protein reduction from 20% to 18% did not affect WG, but a 4% CP reduction (CP20 vs. CP16 (USL+Gly)) significantly decreased WG by 10.19%. Capped dietary starch level significantly prevented WG loss in CP16 groups (P < 0.0001). Weight gain of the CSL+Gly and CSL-Gly diets showed no significant differences compared to the high-protein CP20 diet (1863.38 and 1812.15 vs. 1826.20 g). Capped dietary starch in CP16 diets significantly increased WG in compare to USL (1837.77g vs. 1655.43g; P < 0.0001; Table 4). Glycine supplementation in CP16 diets had no significant effect of WG.

**Table 4:** Effect of dietary treatments on weight gain, feed intake and FCR of male Ross broiler chicks from 15 to 36 days of age.

Treatments^1^			Weight gain (g/bird)	Feed intake (g/bird)	FCR (g/g)
CP 20			1826.20^ab^	2760.33^b^	1.512^c^
CP 18			1767.02^b^	2758.83^bc^	1.561^b^
CP 16	Uncapped starch level	+ Glycine	1640.13^c^	2656.33^c^	1.620^a^
		- Glycine	1670.72^c^	2743.67^bc^	1.642^a^
CP 16	Capped starch level	+ Glycine	1863.38^a^	2873.67^a^	1.563^b^
		- Glycine	1812.15^ab^	2874.83^a^	1.586^b^
SEM			23.11	35.56	0.0076
Main effect	Starch	Uncapped	1655.43	2700.00	1.631
		Capped	1837.77	2874.25	1.575
	Glycine	+	1751.76	2765.00	1.592
		-	1741.43	2809.25	1.614
SEM			18.02	28.08	0.0047
P values	Diet		<0.0001	0.001	<0.0001
	Starch		<0.0001	0.0003	<0.0001
	Glycine		0.69	0.28	0.0041
	Starch × Glycine		0.12	0.29	1

1 CP20: Standard control diet containing 200 g/kg CP; CP18: Low-protein diet containing 180 g/kg CP; CP16 USL+Gly: 160 g/kg CP, uncapped starch level, with glycine supplementation; CP16 USL–Gly: 160 g/kg CP, uncapped starch level, without glycine supplementation; CP16 CSL+Gly: 160 g/kg CP, capped starch level, with glycine supplementation; CP16 CSL–Gly: 160 g/kg CP.

The impact of CP reduction on feed intake exhibited varied outcomes depending on the magnitude of the reduction and specific dietary formulations. A moderate reduction in dietary CP from 20% to 18% had no significant impact on feed intake. However, a more substantial 4% CP reduction, specifically when comparing a 20% CP diet (CP20) against a 16% CP diet supplemented with both USL and Glycine (CP16 (USL+Gly)), resulted in a significant decrease in feed intake (P=0.001; Table 4). Interestingly, the 16% CP diet formulated without USL and Glycine (CP16 USL-Gly) did not show a significant difference in feed intake when compared to the 20% CP diet. These findings underscore that while minor CP adjustments may be tolerated without affecting feed intake, larger reductions can be detrimental unless specific amino acid profiles are maintained. Capped dietary starch level in CP16 diets significantly increased FI in compared to uncapped starch level (P = 0.0003).

Feed conversion ratio significantly increased with every 2% CP reduction from 20% to 18% and to 16% (P < 0.0001). This pronounced statistical significance underscores a critical inverse relationship between dietary CP levels and feed efficiency. Such a consistent rise in FCR across successive 2% decrements in CP suggests that reducing protein below a certain threshold progressively impairs an animal's ability to efficiently convert feed into biomass, likely due to insufficient amino acid availability for optimal growth and metabolic functions.

Capped dietary starch level significantly improved FCR compared to USL (1.567 vs. 1.631; P < 0.0001). Glycine supplementation in CP16 diets improved FCR compared to LP diets with no glycine supplement (1.592 vs. 1.614; P = 0.0041). This statistically significant enhancement indicates that animals receiving glycine-supplemented CP16 diets utilized feed more efficiently, requiring less feed intake to achieve the same growth as those on un-supplemented LP diets.

### Carcass traits

Relative breast weight remained consistent and unaffected by the gradual reduction in CP levels across CP20, CP18, and CP16 treatments, even with the inclusion of USL+Gly supplementation (Table 5). This significant finding indicates that a progressive decrease in dietary CP content, from an initial 20% to 18% and subsequently to 16%, did not compromise the proportional development of breast muscle, a key economic trait. The strategic supplementation with USL and Glycine likely played a crucial role in maintaining adequate amino acid profiles, thereby preventing any adverse impact on protein synthesis and deposition in this vital tissue despite the lower overall CP. However, CSL-Gly diet significantly reduced relative breast weight in compare to CP20, CP18 and CP16 USL diets (P = 0.023), but had no significant difference with CSL+Gly diet. Relative thigh and liver weights were not affected by any factors. Relative abdominal fat weight did not differ between CP20 and CP18 diets but significantly increased with a 4% CP reduction to 16% (P = 0.0338). Relative abdominal fat weight was not affected by dietary starch and glycine levels in the CP16 treatments. Relative pancreas weight significantly decreased with a 4% CP reduction (P = 0.0004), but showed no significant difference between CP20 and CP18 diets. Relative gizzard and proventriculus weight were not affected by CP reduction or glycine levels, but CSL diets had significantly higher relative gizzard and proventriculus weight compared to USL diets (P = 0.0074).

**Table 5:** Effect of dietary treatments on relative weights of breast, thigh, abdominal fat, pancreas, liver and gizzard and proventriculus (% of live weight) at 36 day of age.

Treatments^1^			Breast	Thigh	Abdominal fat pad	Pancreas	Liver	Gizzard & proventriculus
CP 20			26.0^a^	19.2	0.94^b^	0.267^a^	2.55	3.07^ab^
CP 18			26.6^a^	19.5	1.11^ab^	0.268^a^	2.40	3.12^a^
CP 16	Uncapped starch level	+ Glycine	26.4^a^	19.4	1.16^ab^	0.220^b^	2.68	2.99^ab^
		- Glycine	25.7^a^	20.0	1.27^a^	0.227^b^	2.71	2.73^b^
CP 16	Capped starch level	+ Glycine	25.3^ab^	19.3	1.16^ab^	0.218^b^	2.70	3.29^a^
		- Glycine	24.2^b^	19.5	1.30	0.219^b^	2.60	3.23^a^
SEM			0.497	0.277	0.077	0.0097	0.098	0.0119
Main effect	Starch	Uncapped	26.1	19.7	1.21	0.223	2.70	2.86
		Capped	24.8	19.4	1.23	0.218	2.68	3.25
	Glycine	+	25.9	19.4	1.16	0.219	2.69	3.14
		-	25.0	19.8	1.28	0.223	2.69	2.97
SEM			0.379	0.212	0.060	0.007	0.077	0.092
P values	Diet		0.0235	0.41	0.0338	0.0004	0.21	0.0387
	Starch		0.023	0.37	0.86	0.62	0.90	0.0074
	Glycine		0.12	0.19	0.16	0.73	1.00	0.20
	Starch × Glycine		0.75	0.49	0.80	0.81	0.73	0.52

1 CP20: Standard control diet containing 200 g/kg CP; CP18: Low-protein diet containing 180 g/kg CP; CP16 USL+Gly: 160 g/kg CP, uncapped starch level, with glycine supplementation; CP16 USL–Gly: 160 g/kg CP, uncapped starch level, without glycine supplementation; CP16 CSL+Gly: 160 g/kg CP, capped starch level, with glycine supplementation.

### Nutrient utilization

The effects of dietary treatments on parameters of nutrient utilization are shown in Table 6. CP18 and USL-Gly diets significantly showed higher N retention in compare to CP20, USL+Gly, CSL+Gly and CSL-Gly treatments (P < 0.0001), but CP16 USL+Gly showed no difference with CP20 diet. USL treatments showed significantly higher N retention than CSL treatments (P = 0.0002). Similarly, CP18 and USL diets showed higher AME, ME:GE, and AMEn compared to the CP20, USL+Gly, CSL+Gly and CSL-Gly treatments (P < 0.0001). Also, CSL+Gly and CSL-Gly treatments showed lower AME, ME:GE, and AMEn in compare to CP20 diet (P < 0.0001). Capped starch levels in CSL diets led to significantly lower AME, ME:GE, and AMEn compared to USL diet (P < 0.0001).

**Table 6:** Effect of dietary treatments on nutrient utilization [apparent metabolizable energy (AME), metabolizable energy:gross energy ratio (ME:GE), nitrogen (N) retention, N-corrected AME (AMEn)] from 32 to 34 days of age.

Treatment			AME (kcal/kg DM)	ME:GE ratio	N retention (%)	AMEn (kcal/kg DM)
CP 20			3250.54^b^	0.75^b^	70.07^bc^	3228.40^b^
CP 18			3721.36^a^	0.85^a^	81.96^a^	3698.70^a^
CP 16	Uncapped starch level	+ Glycine	3391.92^b^	0.75^bc^	73.21^b^	3374.83^b^
		- Glycine	3757.55^a^	0.83^a^	81.19^a^	3737.92^a^
CP 16	Capped starch level	+ Glycine	2975.77^c^	0.69^c^	70.72^bc^	2958.76^c^
		- Glycine	2970.91^c^	0.70^c^	66.92^c^	2955.65^c^
SEM			80.26	0.018	1.84	79.84
Main effect	Starch	Uncapped	3574.74	0.79	77.20	3556.38
		Capped	2973.34	0.69	68.82	2957.20
	Glycine	+	3183.85	0.72	71.97	3166.79
		-	3364.23	0.76	74.05	3346.78
SEM			56.69	0.013	1.27	56.43
P values	Diet		<0.0001	<0.0001	<0.0001	<0.0001
	Starch		<0.0001	<0.0001	0.0002	<0.0001
	Glycine		0.0359	0.0339	0.26	0.0355
	Starch × Glycine		0.0316	0.0408	0.0038	0.0327

^1^CP20: Standard control diet containing 200 g/kg CP; CP18: Low-protein diet containing 180 g/kg CP; CP16 USL+Gly: 160 g/kg CP, uncapped starch level, with glycine supplementation; CP16 USL–Gly: 160 g/kg CP, uncapped starch level, without glycine supplementation; CP16 CSL+Gly: 160 g/kg CP, capped starch level, with glycine supplementation.

### Serum metabolites

The effects of dietary treatments on serum metabolites are shown in Table 7. The CP18 diet showed significantly higher TG values than CP20 and USL-Gly treatments (P = 0.0239). Serum TG was not affected by starch and glycine in CP16 groups. Crude protein reduction had no effect on LDL, but the capped dietary starch level in the CSL diet showed higher LDL levels than USL diets (P = 0.0439). Serum HDL-C was not affected by CP reduction, but capped dietary starch level significantly increased serum HDL-C compared to the CP20 diet (P = 0.0034). Serum total protein (TP) concentrations remained unaffected despite a gradual reduction in dietary CP levels. Serum TP was significantly increased by capping dietary starch level in CSL diets compared to USL diets (P = 0.0001). Serum uric acid (UA) significantly decreased with CP reduction (P = 0.0004). Also, starch-capped diets showed higher serum UA compared to USL groups (P = 0.0002). CP20 and USL+Gly diets showed lowest blood urea in compare to other diets (P = 0.0004). CSL diets showed higher serum urea than USL diets (P = 0.0028). Glycine supplementation of CP16 diets significantly decreased serum urea (P = 0.0445). Aspartate aminotransferase (AST), alanine aminotransferase (ALT) parameters were not affected by the dietary treatments.

**Table 7:** Effect of dietary treatments on blood serum metabolites of male Ross broiler chicks at 35 of age.

			Blood metabolites^2^							
Treatments^1^			TG mg/dl	LDL mg/dl	HDL-C mg/dl	TP g/l	UA mg/dl	Urea mg/dl	AST u/l	ALT u/l
CP 20			2084.83^c^	34.67^a^	73.67^b^	3.18^b^	4.30^a^	5.67^c^	270.00	49.67
CP 18			2839.33^a^	38.33^a^	76.67^b^	3.27^ab^	3.12^b^	17.83^a^	270.83	34.50
			2482.83^abc^	33.33^ab^	95.00^a^	3.13^b^	2.25^bc^	4.67^c^	281.83	48.33
CP 16	Uncapped starch level	+ Glycine	2482.83^abc^	33.33^ab^	95.00^a^	3.13^b^	2.25^bc^	4.67^c^	281.83	48.33
		- Glycine	2396.17^bc^	27.50^b^	78.50^b^	2.83^c^	2.03^c^	10.83^b^	257.83	40.33
CP 16	Capped starch level	+ Glycine	2457.50^abc^	36.00^a^	95.50^a^	3.45^a^	2.95^bc^	13.00^b^	297.50	48.17
		- Glycine	2512.83^ab^	34.00^a^	96.00^a^	3.47^a^	3.13^b^	14.17^ab^	281.83	57.67
SEM			138.17	2.24	5.00	0.086	0.318	1.56	13.77	9.41
Main effect	Starch	Uncapped	2439.50	30.42	86.75	2.98	2.14	7.75	269.83	44.33
		Capped	2485.17	35.00	95.75	3.46	3.04	13.58	289.67	52.92
	Glycine	+	2470.16	34.67	95.25	3.29	2.60	8.83	289.67	48.25
		-	2454.50	30.75	87.25	3.15	2.58	12.50	269.83	49.00
SEM			94.82	1.51	3.98	0.062	0.14	1.21	9.44	7.30
P values	Diet		0.0239	0.0442	0.0034	0.0001	0.0004	<0.0001	0.45	0.61
	Starch		0.74	0.0439	0.13	<0.0001	0.0002	0.0028	0.15	0.42
	Glycine		0.91	0.0809	0.17	0.12	0.93	0.0445	0.15	0.94
	Starch × Glycine		0.60	0.3791	0.15	0.0848	0.33	0.16	0.76	0.41

1 CP20: Standard control diet containing 200 g/kg CP; CP18: Low-protein diet containing 180 g/kg CP; CP16 USL+Gly: 160 g/kg CP, uncapped starch level, with glycine supplementation; CP16 USL–Gly: 160 g/kg CP, uncapped starch level, without glycine supplementation; CP16 CSL+Gly: 160 g/kg CP, capped starch level, with glycine supplementation.

2 Triglyceride glyceride (TG), LDL, HDL-C, total protein (TP), uric acid (UA), Urea, aspartate aminotransferase (AST), alanine aminotransferase (ALT)

### Starch–protein digestive dynamics

The effects of dietary treatments on Starch–protein digestive dynamics are shown in Table 8, Table 9. Starch digestibility in the DJ and DI was not affected by CP reduction. CSL+Gly and CSL-Gly treatments showed lower starch digestibility in compare to the CP20 diet in DJ and DI (P = 0.0126 and P < 0.0001). Capped starch groups significantly showed lower starch digestibility in DJ and DI than USL groups (P = 0.0115; P = 0.0027; Table 8). Starch disappearance rates in DJ and DI significantly increased with CP reduction (P < 0.0001). Starch disappearance rates of CSL+Gly and CSL-Gly treatments in DJ and DI had no significant differences compared with the CP20 diet. Capped dietary starch level in CP16 diets significantly decreased starch disappearance rates in DJ and DI in compare to USL groups (P < 0.0001). Glycine supplementation of the LP diets significantly decreased starch disappearance rates in DJ and DI (P = 0.0419 and 0.032). Protein digestibility in DJ was reduced by 4% CP reduction (P = 0.0480; Table 9); however, protein digestibility in DI was not affected by CP reduction. Protein digestibility was not affected by starch and glycine levels in LP diets in DJ and DI. Protein disappearance rates in DJ were significantly reduced by CP reduction (P = 0.0007) and significantly reduced in DI by 4% CP reduction (P = 0.0003). Protein disappearance rates in DJ and DI were not affected by starch and glycine treatments. Starch to protein disappearance rate ratios in the DJ and DI significantly increased by CP reduction (P < 0.0001; Table 9). Capping dietary starch level in CP16 diets decreased starch to protein disappearance rate ratios in the DJ tended to be significant (P = 0.069) and significantly decreased in the DI (P<0.0001). Supplementation of glycine in CP16 diets decreased starch to protein disappearance rate ratios in the DJ tended to be significant (P = 0.054), but this parameter in the DI was not affected by glycine in the CP16 diets.

**Table 8:** Effect of dietary treatments on apparent digestibility coefficients and disappearance rates (g/bird/d) of starch in distal jejunum and distal ileum at 35 days of age.

Treatment^1^			Distal jejunum		Distal ileum	
			Digestibility	Disappearance	Digestibility	Disappearance
CP 20			0.90^ab^	41.42^d^	0.95^ab^	42.73^c^
CP 18			0.89^abc^	49.01^c^	0.96^a^	52.96^b^
CP 16	Uncapped starch level	+ Glycine	0.88^bc^	53.34^b^	0.92^bc^	56.14^b^
		- Glycine	0.92^a^	58.21^a^	0.96^a^	60.35^ab^
CP 16	Capped starch level	+ Glycine	0.85^c^	38.93^d^	0.89^c^	41.86^c^
		- Glycine	0.85^c^	40.63^d^	0.90^c^	43.51^c^
SEM			0.017	1.30	0.012	1.12
Main effect	Starch	Uncapped	0.90	55.77	0.94	58.25
		Capped	0.85	39.78	0.89	42.67
	Glycine	+	0.86	46.14	0.91	49.00
		-	0.89	49.42	0.93	51.93
SEM						
P values	Diet		0.0126	<0.0001	<0.0001	<0.0001
	Starch		0.0115	<0.0001	0.0027	<0.0001
	Glycine		0.16	0.0419	0.14	0.032
	Starch × Glycine		0.22	0.31	0.30	0.33

1 CP20: Standard control diet containing 200 g/kg CP; CP18: Low-protein diet containing 180 g/kg CP; CP16 USL+Gly: 160 g/kg CP, uncapped starch level, with glycine supplementation; CP16 USL–Gly: 160 g/kg CP, uncapped starch level, without glycine supplementation; CP16 CSL+Gly: 160 g/kg CP, capped starch level, with glycine supplementation.

**Table 9:** Effect of dietary treatments on apparent digestibility coefficients and disappearance rates (g/bird/D) of protein (N) in distal jejunum and distal ileum and starch:protein disappearance rate ratios in distal jejunum and distal ileum at 36-day post-hatch.

Treatment^1^			Distal jejunum		Distal ileum		Starch to protein disappearance rate ratios	
			Digestibility	Disappearance	Digestibility	Disappearance	Distal jejunum	Distal ileum
CP 20			0.77^a^	22.51^a^	0.77	22.40^a^	1.85^c^	1.93^e^
CP 18			0.64^ab^	16.60^b^	0.79	20.43^ab^	2.97^b^	2.61^c^
CP 16	Uncapped starch level	+ Glycine	0.54^b^	11.45^c^	0.73	15.40^d^	4.60^a^	3.65^a^
		- Glycine	0.74^a^	16.67^b^	0.82	18.57^bc^	3.51^b^	3.25^b^
CP 16	Capped starch level	+ Glycine	0.54^b^	12.79^bc^	0.78	18.41^bc^	3.55^b^	2.29^d^
		- Glycine	0.64^ab^	14.39^bc^	0.74	16.59^cd^	3.12^b^	2.70^c^
SEM			0.059	1.72	0.037	0.98	0.307	0.098
Main effect	Starch	Uncapped	0.64	14.06	0.78	17.00	4.05	3.43
		Capped	0.59	13.59	0.76	17.50	3.34	2.49
	Glycine	+	0.54	12.12	0.75	16.92	4.08	2.97
		-	0.69	15.53	0.78	17.58	3.31	2.95
SEM			0.05	1.25	0.025	0.67	0.26	0.07
P values	Diet		0.0480	0.0007	0.56	0.0003	<0.0001	<0.0001
	Starch		0.48	0.76	0.65	0.60	0.069	<0.0001
	Glycine		0.052	0.07	0.49	0.49	0.054	0.84
	Starch × Glycine		0.47	0.32	0.08	0.02	0.38	0.0004

1 CP20: Standard control diet containing 200 g/kg CP; CP18: Low-protein diet containing 180 g/kg CP; CP16 USL+Gly: 160 g/kg CP, uncapped starch level, with glycine supplementation; CP16 USL–Gly: 160 g/kg CP, uncapped starch level, without glycine supplementation; CP16 CSL+Gly: 160 g/kg CP, capped starch level, with glycine supplementation.

## Discussion

A notable finding of the present study was that, CP18 diet did not affect WG in compared to CP20 diet, but a 4% CP reduction in USL+Gly diet significantly decreased WG by 10.19% (1826.20 g vs.1640.13 g). Previous studies reported that higher amounts and faster absorption rates of uncoated free AAs in LP diets was the main WG depressor factors in LP diets (Namroud et al., 2008; Ospina Rojas et al., 2014; Chrystal et al., 2020a, Chrystal et al., 2020b; Selle et al., 2019; 2022; 2023). According to our results, reduction in dietary CP with encapsulated AAs supplementation did not prevent the negative effect of reducing CP more than 2%.

Feed conversion ratio significantly increased by 3.24% with a CP reduction from 20% to 18% (1.512 vs. 1.561 g/g) and further escalated by 7.14% when CP was reduced from 20% to 16% in the USL+Gly diet (1.512 vs. 1.620 g/g; Table 4). While capped starch level (CSL) in the CP16 diets improved FCR by 3.43% compared to USL groups (1.575 vs. 1.631 g/g), FCR in CSL+Gly and CSL-Gly groups remained significantly higher than the CP20 diet by 3.37% and 4.89% respectively (1.563 and 1.586 g/g vs. 1.512; Table 4). These FCR trends often correlate with increased abdominal fat, as reported by Chrystal et al., 2020a, Chrystal et al., 2020b, a phenomenon widely observed where LP diets significantly increase abdominal fat pad weight in broiler chickens (Fancher & Jensen, 1989; Aletor et al., 2000; Namroud et al., 2009; Darsi et al., 2012; Belloir et al., 2017; Yin et al., 2020; Chrystal et al., 2020a, Chrystal et al., 2020b; Greenhalgh et al., 2020; Strifler et al., 2023; Sadeghi et al., 2024). Consistent with this, the current study found abdominal fat weight significantly increased by a 4% CP reduction, yet was unaffected by dietary starch levels in the CP16 diets, with USL+Gly and USL-Gly groups showing a significant elevation in relative abdominal fat weight by 24.09% and 29.42% compared to the CP20 diet (11.64 and 12.65 g vs. 9.38 g). Therefore, while CSL offered a partial improvement in FCR within lower CP diets, the overarching reduction in dietary crude protein consistently led to a significant increase in both FCR and abdominal fat accumulation.

Fushiki and Iwai, 1989 reported that low-protein diet-fed rats secreted less trypsin and chymotrypsin compared to their high-protein diet-fed counterparts. Furthermore, relative pancreas weight significantly decreased with a 4% CP reduction, an effect not observed with varying starch or glycine levels in the LP diets, an observation indicating that protein is probably a more effective factor on relative pancreas weight than starch.

In the current study, serum TP levels remained unaffected by the gradual reduction of dietary CP from 20% to 18% and subsequently to 16% (USL+Gly) when supplemented with encapsulated amino acids (AAs). Measured serum TP values were 3.18, 3.27, and 3.13 g/l for the 20%, 18%, and 16% CP diets, respectively, indicating no significant variation across treatments. This stability, possibly, suggests that the slowly released encapsulated AAs supplementation of CP-reduced diets effectively prevented the higher serum TP observed in Sadeghi et al. (2024) study by uncoated AAs supplemented LP diets.

Reducing dietary CP levels significantly impacts both dietary starch concentrations and subsequent intestinal starch utilization and its ratio to protein disappearance. Specifically, dietary starch levels systematically increased from 32.64% to 35.58% and further to 41.98% as dietary CP was gradually reduced from CP20 to CP18 and to CP16 USL+Gly, respectively (Table 1). Consequently, compared to the CP20 diet, the intestinal starch disappearance rate significantly increased, rising from 41.42 vs. 49.01 vs. 53.34 g/bird/day in the DJ and from 42.73 vs. 52.96 and 56.14 g/bird/day in the DI for the CP20 vs. CP18 and CP16 USL+Gly diets, respectively. These resulted in a notable increase in the dietary starch-to-protein ratio, escalating from 1.58 in the CP20 diet to 1.95 and 2.69 in the CP18 and CP16 USL+Gly diets, respectively (Table 3), aligning with findings by Selle et al. (2023) who reported increased intestinal starch-to-protein disappearance rate ratios in response to elevated dietary starch-to-protein ratios in LP diets. Intestinal starch to protein disappearance rate ratios in the DJ significantly increased by 60.54% (from 1.85 to 2.97) with a 2% CP reduction (from 20% to 18%) and dramatically by 148.65% (from 1.85 to 4.60) with a 4% CP reduction (from 20% to 16% USL+Gly). These findings underscore a clear compensatory mechanism where reduced dietary protein leads to higher starch inclusion and enhanced intestinal starch utilization relative to protein, impacting overall nutrient assimilation.

The reduction in dietary CP significantly impacted nutrient utilization kinetics, leading to notable increases in starch to protein disappearance rate ratios within the DI segment. Specifically, a 2% CP reduction from 20 to 18% resulted in a significant 35.23% increase in these ratios (1.93 vs. 2.61), while a 4% CP reduction from 20 to 16% (SUL+Gly; Table 9) further escalated them by 89.12% (from 1.93 to 3.65). This observed increment in intestinal starch to protein disappearance rate ratios is likely a contributing factor to the decreased WG reported in USL diets, aligning with findings by Greenhalgh et al. (2020) who documented a linear decrease in WG with increasing starch to protein disappearance rate ratios in the DJ. Moreover, previous research has consistently established a negative correlation between starch to protein disappearance rate ratios in the DJ and WG, alongside a positive correlation with FCR, with similar positive correlations observed for these ratios in the DI segment and FCR (Sydenham et al., 2017; Chrystal et al., 2020a). Consequently, the substantial elevation in intestinal starch to protein disappearance rate ratios following dietary CP reduction is a critical determinant of broiler growth performance and feed efficiency.

Capped dietary starch in LP diets, particularly when supplemented with encapsulated amino acids, offers a promising solution to the inherent challenges posed by high starch levels, thereby improving nutrient utilization and growth performance. Specifically, a capped dietary starch level, formulated closer to a standard CP20 diet, significantly reduced the starch disappearance rate in both the DJ and DI compared to uncapped starch level (USL) groups (P < 0.0001 for both DJ and DI). Furthermore, CSL in these LP diets significantly decreased the starch to protein disappearance rate ratio in the DI and tended to be significant in the DJ compared to USL groups, which is crucial given that a balanced glucose and amino acid ratio at the protein synthesis site is fundamental to efficient protein synthesis and skeletal muscle deposition for growth (Moughan, 2003; Greenhalgh et al., 2020; Liu and Selle, 2015, 2017; Selle et al., 2015, 2019, 2022, 2023). These physiological improvements translated into CSL groups having significantly lower apparent metabolizable energy nitrogen-corrected (AMEn) (2957.20 vs. 3556.38 kcal/kg DM) and significantly higher serum total protein (3.46 vs. 2.98 g/dl) compared to USL groups, possibly reflecting enhanced protein sparing and utilization in CP16 diets. Our findings indicate that capped dietary starch level in LP diets supplemented with encapsulated AAs was responsible for significantly higher WG (1837.77 vs. 1655.43 g) compared to uncapped starch levels, with WG in CSL+Gly and CSL-Gly treatments showing no significant differences compared to the routine high-protein CP20 diet (1863.38 and 1812.15 g vs. 1826.20 g). Ultimately, this approach facilitated a substantial reduction in dietary soybean meal level by 39.22% and 35.01% in CSL+Gly and CSL-Gly treatments, respectively, without any negative effect on WG, demonstrating the successful application of capped starch levels in CP16 diets supplemented with encapsulated amino acids as an effective alternative to conventional high-protein diets.

Despite similar dietary starch levels and significantly lower AMEn received by the CSL treatments compared to CP20 treatment, CSL groups showed significantly higher abdominal fat weight compared to the CP20 treatment. This observation aligns with findings where, in our study, relative abdominal fat weight was not reduced by capped starch levels in LP diets, consistent with Greenhalgh et al. (2020, 2022). Further supporting this, Chrystal et al. (2020a) reported that an AMEn reduction from 3071 to 2871 kcal/kg in an LP diet (CP = 15.6%) did not reduce abdominal fat pad weight. Mechanistically, Fouad and El-Senousey (2014) indicated that LP diets increased abdominal fat weight in broilers by up-regulating malic enzyme mRNA expression and elevating its activity, whereas increasing dietary CP declined abdominal fat pad weight by down-regulating fatty acid synthase enzyme and malic enzyme mRNA expression in the liver of broilers. Moreover, they demonstrated that the application of methionine, lysine, and arginine above recommended levels resulted in down-regulation of fatty acid synthase enzyme and malic enzyme mRNA expression in the liver and decreased abdominal fat pad weight (Fouad and El-Senousey, 2014). Consequently, it appears that the increment in abdominal fat weight in LP diet-fed broiler chickens is likely more dependent on protein/amino acid-related effects, warranting further investigation in future experiments.

Starch digestibility in CSL diets was significantly lower than in the CP20 diet, primarily due to the higher ash content from sand used as an inert filler. Specifically, intestinal starch digestibility coefficients for CSL diets were 0.85 in the DJ and 0.89 in the DI, contrasting with 0.90 (DJ) and 0.95 (DI) observed in the CP20 diet. This reduction is likely attributed to the elevated dietary ash content in CSL diets (137.8 and 150.9 g/kg) compared to CP20 (69.4 g/kg), resulting from the inclusion of sand an inert filler deemed inevitable for capping starch levels in LP diets, as similarly noted by Greenhalgh et al. (2020). Such diminished starch digestibility subsequently led to lower AMEn acquisition than estimated, potentially increasing feed intake (FI), reducing skeletal muscle deposition, and consequently affecting FCR, though intriguingly, WG in the CSL treatments remained unaffected. Furthermore, CSL+Gly and CSL-Gly treatments exhibited similar WG to CP20 treatments (Table 4) and shared comparable estimated starch-to-lysine ratios despite varying dietary starch-to-protein levels (Table 3). These observations suggest that the dietary starch-to-lysine ratio, as proposed by Hartono et al. (2014), could offer a more accurate and practical metric for managing dietary glucose-to-amino acid digestive dynamics and postprandial synchronism in LP diets, warranting further dedicated research (Table 5).

The incorporation of higher levels of free amino acids in LP diets has been consistently linked to ammonia toxicity and subsequent growth suppression in broilers (Selle et al., 2022), a condition known to overwhelm the hepatic detoxification capacity of the liver (Selle et al., 2023). Considering that ammonia detoxification necessitates adequate glycine for uric acid synthesis (Salway, 2018), with up to 49.2% of dietary glycine utilized for this purpose (Selle et al., 2021), dietary glycine supplementation has been explored as a potential mitigant. While some studies have reported that supplemental glycine decreased blood ammonia and enhanced growth performance (Ospina Rojas et al., 2014; Hofmann et al., 2019), other research found no such improvement with glycine supplementation in LP diets (Van Harn et al., 2018) or failed to prevent growth depression when CP reduction exceeded 3% (Wang et al., 2020). Consistent with some of these varied findings, our results indicated no significant effect of glycine supplementation on broiler WG and FI, nor was there a significant interaction between glycine and starch levels on WG, FI, or FCR. Nevertheless, as a main effect, glycine supplementation of LP diets significantly improved FCR by 1.36% (P=0.0041), a positive outcome potentially attributed to a 29.36% lower blood urea (P=0.0445), significantly reduced starch disappearance rates in DI (P=0.0419) and DJ (P=0.032), and a consequent lowering of AMEn (P=0.0335) in the glycine-supplemented LP diets. These findings suggest a nuanced role for glycine supplementation in LP broiler diets, primarily enhancing feed efficiency through metabolic adjustments rather than directly impacting growth rate.

## Conclusion

Supplementation of the LP diets with slow-release form of the AAs could not prevent negative effect of LP diets on WG and FCR, solely. Capped dietary starch level in LP diets supplemented with encapsulated AAs significantly improved WG and FCR. Nevertheless, higher abdominal fat pad weight in LP diets was complicated to solve and needs to be more investigated. Glycine supplementation had no significant effect on WG in the LP diet fed broilers, but significantly improved FCR.

## Funding sources

This research did not receive any specific grant from funding agencies in the public, commercial, or not-for-profit sectors.

## CRediT authorship contribution statement

**Ramin Shahabi:** Writing – original draft, Formal analysis, Data curation. **Hassan Darmani Kuhi:** Writing – review & editing, Validation, Supervision, Project administration. **Mohammad Roostaie Ali-Mehr:** Methodology, Investigation, Data curation. **Houshang Lotfollahian:** Investigation, Formal analysis, Data curation.

## Disclosures

The authors declare that they have no known competing financial interests or personal relationships that could have appeared to influence the work reported in this paper.
